# Sex Trafficking Myth Reduction: Evaluating an Educational Approach to Reducing Victim Blaming and Increasing Victim Empathy

**DOI:** 10.1002/bsl.70034

**Published:** 2025-12-20

**Authors:** Dara Mojtahedi, Gemma Hewitt, Sophie Fitton

**Affiliations:** ^1^ Department of Social and Psychological Sciences, School of Human and Health Sciences University of Huddersfield Huddersfield UK

**Keywords:** attitudes, intervention, myths, sex trafficking, sexual exploitation, victim blaming, victim empathy

## Abstract

This study examined the effectiveness of a brief educational intervention designed to reduce sex trafficking (ST) myth acceptance. Using a 2 × 2 mixed design, participants (*N* = 189) viewed either an educational video addressing common ST myths or a control video on human memory. Measures of ST myth acceptance and victim empathy were collected before, immediately after, and 1 month following the intervention. Participants also evaluated a vignette describing an alleged ST case and responded to items assessing empathy, victim blaming, and perceptions of the defendant's guilt. The intervention did not significantly reduce ST myth acceptance or influence vignette‐based judgements, which may reflect a ceiling effect given participants' already supportive baseline attitudes. However, a protective effect emerged over time: participants in the intervention condition maintained supportive victim attitudes at follow‐up, whereas control participants demonstrated increased victim blaming and decreased empathy.

## 1 Introduction

Harmful misconceptions about sexual violence are pervasive and play a significant role in shaping societal responses to victims (Krahé 2016). Primarily examined within the context of sexual assault and rape, the term *rape myths* have been used to capture problematic and inaccurate beliefs about rape that function to shift blame onto victims (e.g., ‘women who dress provocatively are asking for it’), remove culpability from offenders (e.g., ‘he couldn't help himself’), and/or deny the offence altogether (e.g., ‘women lie about rape’; Burt 1980; Lilley, Willmott, Mojtahedi, and Labhardt 2023). Alarmingly, there is growing evidence that rape myths negatively influence criminal justice processes: Researchers argue that rape myth acceptance contributes to the high attrition rates observed in rape cases (Stewart et al. 2024; Willmott et al. 2021), affects how victims are treated by professionals such as police interviewers (Shaw et al. 2017), and shapes juror decision‐making, such that jurors who endorse rape myths are more likely to discredit a complainant's testimony and believe that of the defendant (Lilley, Willmott, and Mojtahedi 2023; Parsons and Mojtahedi 2022; Richardson et al. 2025; Willmott 2018). Despite extensive scholarship on rape myths, the broader landscape of sexual violence myths, particularly those concerning sex trafficking (ST), has received limited empirical attention—a research gap that the following study addresses.

### 1.1 Sex Trafficking and It's Misconceptions

ST is a global issue and is entrenched in most societies (United Nations Office on Drugs and Crime 2016). In 2024, 1546 females were referred to the British Home Office for ST (Home Office 2025), while in the United States, law enforcement agencies reported approximately 2390 cases of ST in 2022 (Remrey and Gardner 2024). These figures are likely to be underestimations, as many victims remain unidentified (Kara 2009; Long and Dowdell 2018), and some identified victims may be classified under alternative offence categories (Digidiki et al. 2016; O’Brien 2009). The impact of ST on victims is multifaceted and enduring: victims are subjected to emotional, psychological, sexual, and physical abuse during their exploitation, and many report long‐term effects on their mental, reproductive, and physical health (Lederer and Wetzel 2014; Reid and Piquero 2014).

There remains a general lack of public understanding surrounding ST in comparison to other gender‐based crimes (Cunningham and Cromer 2016; Digidiki et al. 2016). Consequently, acceptance of ST myths has been observed among both laypeople (Cunningham and Cromer 2016; Farrell and Pfeffer 2014; Q. C. Miller et al. 2023) and professionals in victim‐facing roles (V. R. Anderson et al. 2017; Hartinger‐Saunders et al. 2017; Renzetti et al. 2015; Viergever et al. 2015). These myths primarily concern beliefs around what counts as ‘real’ ST, as well as beliefs that victims' are complicit in the sexual transactions.

Media portrayals often reinforce stereotypical beliefs that ‘real’ ST typically occurs in foreign or developing countries (Todres 2009). As a result, many individuals in Western societies fail to recognise ST as a local problem and are less likely to identify cases that do not align with stereotypical images of a victim (Houston‐Kolnik et al. 2016). One survey of mandated reporters (e.g., teachers, healthcare workers and social workers) in the United States found that 25% of respondents did not believe that ST occurred in their communities, 21% believed that most victims came from other nations, and 10% thought that ST was ‘blown out of proportion’ in their country (Hartinger‐Saunders et al. 2017).

Another stereotype surrounding ‘real’ victims, stemming from just‐world biases (see Van den Bos and Maas 2009), concerns the expectation of moral innocence (Vonderhaar and Carmody 2015). Individuals holding such expectations are less likely to recognise a complainant as a victim if they are perceived to have engaged in other illegal activities (Birks and Gardner 2019; Buckley 2009). This is particularly problematic in the context of ST, as these victims are at increased risk of substance misuse and involvement in prostitution (Koegler et al. 2022; Langton et al. 2022).

Victim‐blaming of ST victims primarily stems from two myths related to exploitation and control. While it is not uncommon for victims to receive some form of payment from their exploiters as a means of manipulation and control (Kara 2009), such payments are often misinterpreted as evidence of complicity or consent (Cunningham and Cromer 2016; Digidiki and Baka 2020; Farley et al. 2004). Hartinger‐Saunders et al. (2017) found that 57% of mandated reporters believed that victims of ST had willingly chosen to prostitute themselves. Another prevalent myth is that victims could simply end their exploitation by leaving their traffickers (Houston‐Kolnik et al. 2016). In reality, victims face complex trauma, coercive control, and structural barriers that make escape exceedingly difficult (Zimmerman et al. 2011). Many also develop mistrust towards the police, often due to misunderstandings about their rights and fears of punishment (Kara 2009; Langton et al. 2022).

### 1.2 Consequences of ST Myth Acceptance

Emerging research has demonstrated the risks that ST myth acceptance poses to victims and the criminal justice system. At a societal level, such myths can influence individuals' empathy towards victims (Mojtahedi et al. 2024) and their support for victim‐focused policies (Clements et al. 2006; Houston‐Kolnik et al. 2016). At a procedural level, ST myths can affect the behaviour of professionals who interact with victims. For example, evidence suggests that acceptance of these myths can compromise the ability of victim‐facing professionals to identify victims (Menaker and Franklin 2015). Interviews with ST survivors also reveal that some victims experience ST myth narratives during their interviews with police and healthcare workers (i.e., victim blaming language), which ultimately deters them from seeking further help (Rajaram and Tidball 2018). ST myths can also impact the outcome of court cases. In a mock juror experiment, Stevens et al. (2024) found a relationship between jurors' endorsement of ST myths and their assessments of complainant and defendant credibility. Specifically, participants who endorsed victim‐blaming myths were more likely to believe the defendant's testimony, while endorsement of myths concerning what constitutes ‘real’ ST was associated with reduced complainant credibility. These myths did not, however, appear to influence the final verdict—a result the authors suggested might reflect a ceiling effect (i.e., a low frequency of not‐guilty verdicts).

Understanding where ST myth acceptance comes from is a fundamental step to reducing its impact. Mojtahedi et al. (2024) examined public attitudes towards ST victims in a two‐part study. In study one, the authors found that participants who were less knowledgeable about ST displayed less empathy towards victims. Findings from study two suggested that that negative attitudes towards ST victims (including myth acceptance) stemmed from internal biases against women. Specifically, participants exhibiting higher levels of hostile sexism expressed significantly lower empathy towards victims and were more likely to blame them for their own abuse—a relationship also observed in other forms of gender‐based violence (Abrams et al. 2003). Mojtahedi and colleagues' findings suggest that ST myth acceptance can stem from both inaccurate knowledge about the crime, as well as pre‐existing biases towards victim groups. Building on these insights, the present study explores whether an educational intervention can effectively reduce the prevalence and impact of ST myths, or whether entrenched biases might attenuate the effects of such a knowledge‐based approach.

### 1.3 Myth Reduction Interventions

There is some evidence to suggest that educational interventions—both public‐facing (e.g., awareness campaigns; see U.S. Government Accountability Office, 2023) and person‐ or group‐focused (e.g., training for medical and legal professionals; see Beck et al. 2015; Farrell and Pfeffer 2014)—have the potential to improve public knowledge and awareness surrounding ST. To the authors' knowledge, only Q. C. Miller et al. (2023) have empirically tested the efficacy of educational interventions in reducing ST myths. Their findings indicated that myth acceptance decreased following intervention, with fact‐based training demonstrating a greater impact than a story‐based approach. However, the authors did not explore whether these interventions would influence behaviour in applied settings, such as juror decision‐making in ST cases.

Given the paucity of research on interventions designed to reduce ST myths, further insights can be drawn from the extensive literature on rape myth reduction. Whilst a considerable body of research indicates that educational interventions can reduce endorsement of rape myths (L. A. Anderson and Whiston 2005; Hudspith et al. 2023, 2024; Leverick 2020; Reddy et al. 2022), some studies have reported limited or inconsistent effects (see FitzGerald et al. 2019, for a review). Such inconsistencies may stem from sampling differences—for instance, samples with low baseline myth acceptance are less likely to demonstrate significant reductions post‐intervention due to a floor effect (Meier 2022)—as well as from variations in the quality and design of intervention approaches.

Drawing on a systematic review of rape myth reduction interventions, Hudspith et al. (2023) concluded that effective interventions tend to be delivered in video format by a presenter regarded as credible on the subject, structured to challenge common myths using factual information and can be relatively brief (also see Anderson and Whiston 2005; Hudspith et al. 2024; Leverick 2020). However, some scholars have questioned the reliability of the ‘myths versus facts’ approach in dispelling misconceptions. Krahé (2025) cautioned that presenting individuals with myths alongside factual corrections might prove counterproductive, as participants could later misremember the myths as facts. This argument draws on the ‘illusory truth effect’, a well‐documented cognitive bias whereby individuals are more likely to perceive information as true simply because they have encountered it before (see Udry and Barber 2024). Although this phenomenon has yet to be evidenced in the context of sexual violence, similar ‘backfire effects’ have been reported in attempts to challenge political (Nyhan and Reifler 2010) and mental health (Dobson and Rose 2022) misconceptions using a myths‐versus‐facts approach.

## 2 Present Study

ST myths have the potential to undermine the investigation and prosecution of ST offences, as well as the treatment and protection afforded to victims (Farrell and Pfeffer 2014; Rajaram and Tidball 2018). Emerging research suggests that appropriate training can reduce myth acceptance (Q. C. Miller et al. 2023), enhance the practice of professionals working with victims (Öztürk 2021), and mitigate bias during juror decision‐making (see Hudspith et al. 2024, for a review). However, much of this evidence derives from interventions addressing other forms of gender‐based violence, with comparatively little research evaluating strategies specifically aimed at reducing ST myths.

The present study sought to address this gap by examining the impact of an educational intervention video on attitudes towards ST, guided by two key objectives. First, the study investigated whether the intervention reduced ST myth acceptance. Based on existing evidence, it was hypothesised that participants who viewed the educational intervention video would report a greater reduction in ST myth acceptance and greater increase empathy towards victims, compared to participants who did not view the educational intervention video (Hypothesis 1).

The second objective assessed the influence of the educational intervention on participants' reactions to a specific ST case presented in a vignette, focussing on three outcomes: victim empathy, victim blame, and certainty of defendant guilt. It was hypothesised that participants who viewed the educational intervention video would report higher levels of victim empathy (Hypothesis 2) and defendant guilt certainty (Hypothesis 3), and lower levels of victim blame (Hypothesis 4), compared with participants who did not view the educational intervention video.

## 3 Methods

### 3.1 Participants

An a priori power analysis was conducted for the most sample‐intensive analysis in the study (a mixed factorial ANOVA). Using a significance criterion of *α* = 0.05 and power = 0.80, the minimum sample size required to detect a medium effect (*f* = 0.25, as reported in Reddy et al. 2022) was 54 participants. However, the authors elected to exceed this threshold, given that some previous studies have reported smaller effects (see Hudspith et al. 2023, for a review).

An opportunity sampling approach was employed. A link to the online experiment was disseminated via social media platforms and online community groups. A total of 203 participants completed the experiment; however, 13 responses were removed for failing attention or manipulation checks, resulting in a final usable sample of 189 participants (152 female). Participants' ages ranged from 21 to 74 with a mean of 37.58 years (SD = 16.32).

### 3.2 Procedure and Design

The study received ethical approval from the authors' institutional research ethics committee. All participants provided informed consent prior to commencing the online experiment.

Participants first completed a battery of questionnaires, including demographic questions and measures of attitudes towards ST (see below). They were then randomly assigned to one of two intervention conditions. Participants in the educational intervention condition viewed an educational video designed to refute common ST misconceptions and subsequently completed a brief manipulation check in which they described the video content. Participants in the control condition completed the same process but viewed an unrelated video discussing the accuracy of human memory.

Following the intervention, participants completed the ST attitude questionnaires for a second time and were then presented with a vignette describing an alleged case of ST. After reading the vignette, participants completed a questionnaire assessing their attitudes towards the case and the alleged victim.

Participants were invited to complete a follow‐up ST attitude questionnaire 4 weeks later; however, only 36 responses were received. Consequently, the main analyses of intervention effects focused on pre‐ and post‐intervention scores to ensure sufficient statistical power. A supplementary longitudinal analysis using the reduced follow‐up sample was also conducted.

### 3.3 Materials

#### 3.3.1 Sex Trafficking Attitudes

The Sex Trafficking Attitudes Scale (STAS; Houston‐Kolnik et al. 2016) is a 27‐item instrument measuring six dimensions of beliefs about ST. The present study used the four subscales pertaining to myths about ST victims: *Attitudes Toward Ability to Leave Sex Trafficking* (*Leave*; five items; Cronbach's *α* = 0.805) measures individuals' understanding of the challenges victims face in escaping their exploiters, with lower scores indicating greater victim‐blaming attitudes. *Knowledge About Sex Trafficking* (*Knowledge*; four items; Cronbach's *α* = 0.839) assesses awareness of the various forms of ST; lower scores reflect more constrained and stereotypical beliefs about what constitutes ‘real’ ST. *Attitudes Toward Helping Survivors* (*Help*; three items; Cronbach's *α* = 0.901) reflects participants' perspectives on how ST survivors should be supported. Higher scores represent empowering attitudes, whereas lower scores reflect paternalistic beliefs rooted in the misconception that all victims are incapable of making safe life choices. *Empathetic Reactions Toward Sex Trafficking* (*Empathy*; five items; Cronbach's *α* = 0.895) measures affective responses towards victims of ST. Although this subscale does not directly assess a specific myth, it was included given the well‐established association between ST myth acceptance and reduced concern for victims (Mojtahedi et al. 2024). Participants responded to each item using a six‐point scale (1 = *strongly disagree* to 6 = *strongly agree*), items within each subscale were averaged to produce total scores which ranged from 1 to 6 with higher scores reflecting more positive victim attitudes.

#### 3.3.2 Sex Trafficking Vignette

The ST vignette from Mojtahedi et al. (2024) was adapted for use in the present study (see Supporting Information S1). The original vignette, based on a real case, described an adult female coerced by her partner into commercial sex work for over a year, after which she escaped and reported him to the police. In the adapted version, the victim did not escape but reported her partner following her own arrest for alleged prostitution. The revised vignette also included a statement from the partner asserting that the victim was complicit in the sex work. These adaptations allowed assessment of participants' judgements of guilt in a contested‐account scenario. Following the vignette, participants answered a series of questions assessing their attitudes towards the case, along with an attention‐check question requiring them to describe the substances taken by the victim.

##### 3.3.2.1 Victim Empathy

Ten items (Cronbach's *α* = 0.734) adapted from Franklin and Garza (2021) measured participants' empathy towards the victim (e.g., ‘I feel sorry for [victim] and her problems’). Items were rated on a seven‐point scale (1 = *strongly disagree* to 7 = *strongly agree*), with higher mean scores indicating greater empathy.

##### 3.3.2.2 Victim Blaming

Four items (Cronbach's *α* = 0.851) adapted from Brown and Testa (2008) assessed victim‐blaming attitudes (e.g., ‘To what extent is [victim] to blame for what happened?’). Items were rated on a nine‐point scale (1 = *not at all* to 9 = *to a great extent*), with higher mean scores indicating stronger victim‐blaming tendencies.

##### 3.3.2.3 Verdict and Guilt Certainty

Participants were asked, ‘If this case went to trial and you were serving as a juror, how would you find the defendant on the charge of “Causing a person to engage in sexual activity without consent” under Section 4 of the Sexual Offences Act 2003?’ (a definition of the legislation was provided). Participants gave a categorical verdict (*guilty/not guilty*) and rated their confidence on a 10‐point scale (1 = *not confident at all* to 10 = *extremely confident*). A continuous measure of *guilt certainty* was created by multiplying the verdict decision (−1 = *not guilty*; 1 = *guilty*) by the confidence score, producing a scale from −10 (high certainty in a not‐guilty verdict) to +10 (high certainty in a guilty verdict). This approach has been widely used in jury decision‐making research (e.g., M. K. Miller and Bornstein 2006; M. K. Miller et al. 2011) to capture varying degrees of confidence in verdicts.

#### 3.3.3 Educational Intervention

The educational intervention (available at: https://youtu.be/v5RJc1PNM3E) was designed in line with recommendations from Hudspith et al. (2023). The intervention consisted of a 6‐minute, twenty‐one‐second video in which factual information was presented by an individual deemed credible on the topic (the presenter was introduced as an Associate Professor in Forensic Psychology with research expertise in criminal justice and ST). Based on the findings from Q. C. Miller et al. (2023), the intervention presented *fact‐based* information rather than narrative accounts. The content addressed common myths identified in previous research (e.g., Houston‐Kolnik et al. 2016), including: the prevalence of ST in Western countries; the difficulties victims face in leaving exploitative relationships; the various methods traffickers use to control and manipulate victims; the vulnerabilities that increase susceptibility to exploitation; and the misconception that receiving payment implies consent or complicity. To mitigate the risk of an *illusory truth effect* (see Krahé 2025), the video did not explicitly present the myths themselves but instead provided factual statements that implicitly refuted them. Participants in the control condition viewed a video identical in format, length, and presenter, but focused on an unrelated topic (eyewitness memory). A manipulation check question was included asked participants to ‘describe a key message from the video’ using an open‐dialogue box. Careful inspection of the qualitative responses revealed that participants retained the educational information from the interventions.

To assess the perceived accuracy and credibility of the intervention content, participants rated their agreement (1 = *strongly disagree* to 5 = *strongly agree*) with two statements: ‘The information presented in the video seemed accurate’ and ‘The presenter in the video seemed credible on the topic’.

### 3.4 Analyses

All statistical analyses were conducted using SPSS version 26.0 (IBM Corporation, Armonk, NY, USA) for Windows. For ANOVA and *t*‐tests, skewness and kurtosis values were inspected to ensure that the data were appropriately distributed. Effect sizes were interpreted in accordance with Cohen's (1988) guidelines.

## 4 Results

### 4.1 Descriptive Data on ST Attitudes

Descriptive statistics for all attitudinal and vignette responses were first examined to assess the sample's attitudes towards ST victims (see Table 1). Participants demonstrated significantly higher scores on the Sex Trafficking Attitudes Scale (STAS)—indicating lower acceptance of ST myths—compared with those reported in previous research (e.g., Mojtahedi et al. 2024; see Table 2). Guilt judgements were also skewed, with most participants in both the educational intervention (95.7%) and control (93.3%) groups finding the defendant guilty of ST.

**TABLE 1:** Mean and standard deviation scores for ST attitudes and vignette responses.

	Range	Control intervention		Educational intervention	
		Pre‐intervention	Post‐intervention	Pre‐intervention	Post‐intervention
Leave	2 to 6	5.3 (0.81)	5.05 (0.92)	5.13 (0.94)	5.24 (0.82)
Empathy	2 to 6	5.4 (0.71)	5.23 (0.72)	5.21 (0.93)	5.27 (0.93)
Knowledge	2 to 6	5.38 (0.72)	5.36 (0.74)	5.24 (0.89)	5.41 (0.9)
Help	1 to 6	3.79 (1.34)	3.68 (1.41)	3.63 (1.39)	3.47 (1.46)
Victim blaming (vignette)	1 to 7		2.88 (1.45)		2.63 (1.53)
Empathy (vignette)	2.1 to 7		5.81 (0.99)		5.92 (0.96)
Guilt certainty	−8 to 10		6.99 (3.69)		7.47 (3.31)

**TABLE 2:** Sample comparison with Mojtahedi et al. (2024).

Variable	Current sample (pre‐intervention)	Mojtahedi et al. (2024)	*d*
Leave	5.2 (0.88)	3.31 (1.08)	1.85
Knowledge	5.31 (0.82)	4.56 (0.88)	0.87
Empathy	5.3 (0.84)	4.18 (0.86)	1.31
Help	3.7 (1.35)	3.11 (1.05)	0.51

*Note: p* < 0.001 for all comparisons.

### 4.2 Short‐Term Intervention Effects

Participants who viewed the educational intervention strongly agreed that the presenter was credible (Mdn = 5, IQR = 1) and that the information presented was accurate (Mdn = 5, IQR = 1). A series of 2 × 2 mixed analyses of variance (ANOVAs) were conducted to compare ST attitude scores (Leave, Empathy, Knowledge, and Help) between participants in the control and educational intervention groups, pre‐ and post‐intervention. Inferential statistics for all main and interaction effects are presented in Table 3.

**TABLE 3:** Inferential properties for between and within group comparisons.

	Intervention condition	Re‐test effect	Interaction
ST attitude scales			
Leave	*F* (1,182) = 0.01, *p* = 0.906, *ηp* ^2^ < 0.001	*F* (1,182) = 2.02, *p* = 0.157, *ηp* ^2^ = 0.01	*F* (1,182) = 14.78, *p* < 0.001, *ηp* ^2^ = 0.08
Empathy	*F* (1,182) = 0.5, *p* = 0.483, *ηp* ^2^ = 0.003	*F* (1,182) = 10.75, *p* = 0.001, *ηp* ^2^ = 0.06	*F* (1,182) = 10.75, *p* < 0.001, *ηp* ^2^ = 0.06
Knowledge	*F* (1,182) = 0.16, *p* = 0.694, *ηp* ^2^ = 0.001	*F* (1,182) = 1.92, *p* = 0.168, *ηp* ^2^ = 0.01	*F* (1,182) = 2.6, *p* = 0.109, *ηp* ^2^ = 0.01
Help	*F* (1,182) = 0.89, *p* = 0.347, *ηp* ^2^ = 0.01	*F* (1,182) = 5.12, *p* = 0.025, *ηp* ^2^ = 0.03	*F* (1,182) = 0.23, *p* = 0.632, *ηp* ^2^ = 0.001
Vignette response			
Victim empathy	*t* (184) = −0.83, *p* = 0.204, *d* = 0.12		
Victim blame	*t* (184) = 1.11, *p* = 0.133, *d* = 0.16		
Guilt certainty	*t* (179) = 0.93, *p* = 0.177, *d* = 0.14		

**FIGURE 1:**
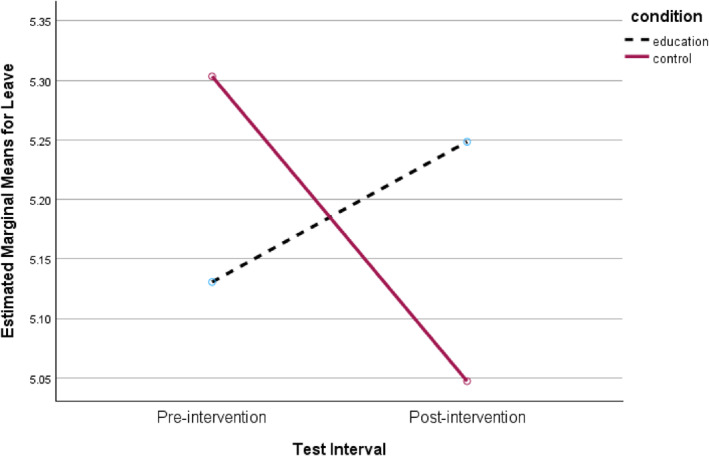
Interaction between intervention group and test interval for Leave scores.

**FIGURE 2:**
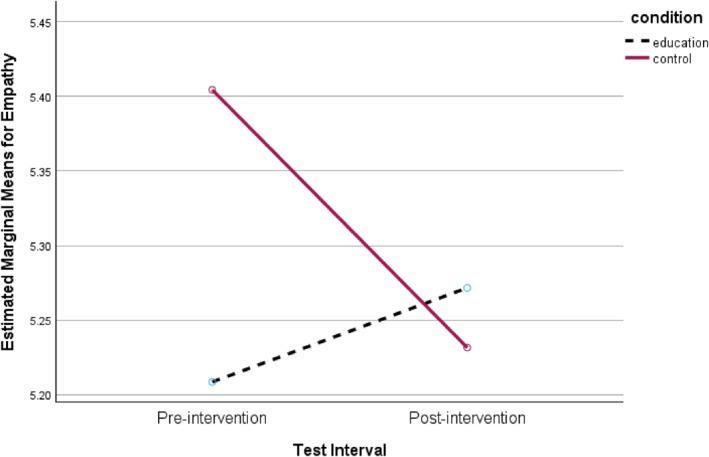
Interaction between intervention group and test interval for Empathy scores.

No significant main effects were observed for intervention condition or re‐test effects. Although the within‐subjects effect for Help scores fell below the conventional significance threshold of *p* < 0.05 (*p* = 0.025), this result did not remain statistically significant following Bonferroni correction (adjusted *α* = 0.013). Two significant interaction effects were, however, observed for the Leave and Empathy subscales, both with moderate effect sizes. As illustrated in Figures 1 and 2, participants who viewed the educational intervention reported marginally higher (though negligible) Leave and Empathy scores at re‐test, whereas those in the control condition exhibited decreases in these scores.

For the vignette responses, independent‐samples *t* tests indicated that the educational intervention did not significantly influence victim empathy, victim blaming, or defendant guilt certainty (see Table 3).

### 4.3 Longitudinal Intervention Effects

Given the observed short‐term interaction effects for Empathy and Leave scores, two additional 2 × 3 mixed ANOVAs were conducted on a reduced cohort of participants (control: *n* = 19; intervention: *n* = 17) who completed the 1‐month follow‐up questionnaire to examine potential longitudinal effects.

For Leave scores, there were no significant main effects of intervention condition, *F*(1, 34) = 0.003, *p* = 0.956, *ηp* ^2^ < 0.001, or test interval, *F*(1, 34) = 0.04, *p* = 0.834, *ηp* ^2^ = 0.001, nor was there a significant interaction, *F*(1, 34) = 0.004, *p* = 0.953, *ηp* ^2^ < 0.001.

**FIGURE 3:**
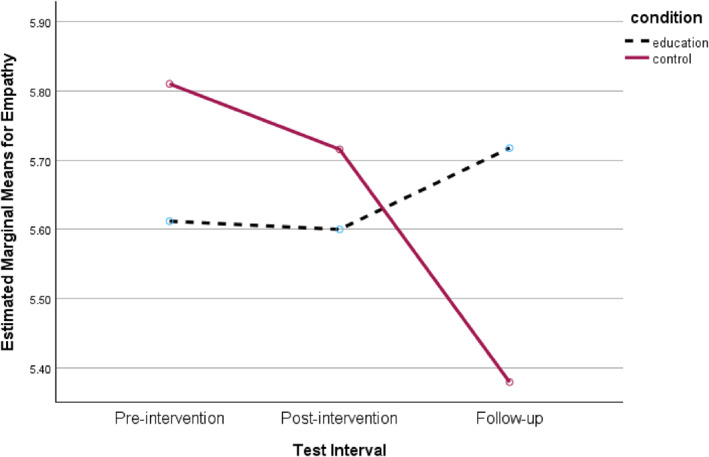
Interaction between intervention group and test interval for Empathy scores.

For Empathy scores, there were no significant main effects of intervention condition, *F*(1, 34) = 0.27, *p* = 0.609, *ηp* ^2^ = 0.01, or test interval, *F*(1.63, 34) = 2.66, *p* = 0.089, *ηp* ^2^ = 0.07. However, the interaction effect was statistically significant, *F*(1.63, 34) = 8.07, *p* = 0.002, *ηp* ^2^ = 0.19, representing a large effect size. As depicted in Figure 3, participants who received the educational intervention demonstrated small but sustained increases in Empathy scores 1 month after the experiment, whereas participants in the control condition showed declines over the same period.

## 5 Discussion

The present study examined the impact of an educational intervention video on attitudes towards ST, with a particular focus on ST myth endorsement. Overall, participants demonstrated highly supportive attitudes towards ST victims. Mean responses indicated that most participants were empathetic towards victims, knowledgeable about the different forms of ST, and unlikely to attribute blame to victims. The only exception to this pattern concerned attitudes towards helping survivors, where participants appeared divided between adopting an empowering approach (supporting survivors to take control of their lives) and a paternalistic one (making decisions on behalf of survivors for their own good). This division may reflect a perception that both approaches can be helpful in different contexts. A large majority of participants also favoured convicting the defendant in the vignette for ST offences. While these findings could be interpreted positively, suggesting that members of the public are relatively well‐informed and dismissive of harmful myths, it should be noted that participants in this study scored more favourably than those reported in previous studies using the same measures (e.g., Mojtahedi et al. 2024). This suggests that the current sample may not be representative of wider public attitudes. Accordingly, the findings should be interpreted with caution.

Participants' attitudes towards ST did not significantly improve following exposure to the educational intervention. Although small positive shifts were observed across most attitudinal measures, these changes were negligible. Consequently, the first hypothesis was not supported. Similarly, the intervention did not significantly influence participants' responses to the ST vignette in terms of victim empathy, victim blaming, or defendant guilt certainty, meaning that hypotheses two, three, and four were also unsupported. These findings contrast with those of Q. C. Miller et al. (2023), who observed significant reductions in ST myth endorsement following an educational intervention, as well as with a broader body of literature on rape myth reduction interventions that have demonstrated positive attitudinal change (e.g., L. A. Anderson and Whiston 2005; Leverick 2020; Reddy et al. 2022).

There are several possible explanations for these contrasting findings. One interpretation is that the present intervention was not optimally designed to target the specific myths or cognitive mechanisms underpinning ST attitudes. Although it was modelled on evidence‐based recommendations, the intervention employed a unidimensional, knowledge‐based approach through a brief educational video. Prior research suggests that the many effective myth‐reduction interventions incorporate multiple components, such as facilitated discussion (Elias‐Lambert and Black 2016; Palm Reed et al. 2015), interactive exercises (Forst et al. 1996; Salazar et al. 2014), and perspective‐taking activities to promote empathy (O’Donohue et al. 2003; Schewe and O’Donohue 1996). The present intervention also differed in duration; at less than 7 minutes, it was considerably shorter than the 42‐min fact‐based video used by Q. C. Miller et al. (2023), which may have contributed to the smaller observed effects.

It is also plausible that ST myths are sustained through cognitive biases such as confirmation bias (as observed in rape myth acceptance; see Süssenbach et al. 2017) and motivated reasoning (Ananyeva et al. 2024), which may not be easily altered through information‐based approaches alone. Nevertheless, the majority of participants rated the intervention content as accurate and the presenter as credible. Thus, a more compelling explanation for the null findings is that participants' baseline attitudes were already strongly pro‐victim, leaving little room for improvement—a ceiling effect that likely limited observable intervention impact.

Despite the absence of significant attitudinal change, the intervention appeared to exert a protective effect on scores for the Leave and Empathy subscales when compared with control group participants. While the educational video did not produce measurable improvements in ST attitudes, control participants reported reductions in empathy towards victims and more misconceptions regarding victims' ability to leave abusive situations. Follow‐up analyses suggested that these differences persisted 1 month later: empathy scores increased slightly among participants who viewed the educational intervention but declined further among control participants. Although no clear theoretical explanation accounts for the observed decline in the control group, a plausible methodological interpretation is that participants became less influenced by social desirability effects upon retesting. Nonetheless, the results indicate that even a brief educational intervention can help buffer against the deterioration of empathetic or inaccurate attitudes over time.

Such a protective effect against ST myth acceptance holds potential relevance for criminal justice contexts. Experimental simulations have shown that jurors can rely on rape myths to justify verdicts (Richardson et al. 2025), and that such reasoning can shape the views and decisions of other jurors during deliberations (Lively 2017). Similar biases may arise among professionals in victim‐facing roles, as evidenced by persistent rape myth acceptance within policing cultures (Garza and Franklin 2021; Gekoski et al. 2024). Although the present study did not examine interactions between intervention and subsequent ST myth exposure, the findings suggest that educational interventions could be valuable for reducing individuals' susceptibility to ST myths in professional or judicial settings. This highlights an important avenue for future research.

While the present study adds to the limited empirical evidence on ST myths, several limitations should be acknowledged. First, as noted, the representativeness of the sample is questionable. Participants exhibited overwhelmingly pro‐victim baseline attitudes, which contrast with previous findings (e.g., Q. C. Miller et al. 2023; Mojtahedi et al. 2024). It is possible that a more diverse sample—encompassing a broader spectrum of beliefs—would yield different results and potentially reveal stronger intervention effects.

Second, the study experienced higher‐than‐expected attrition at the 1‐month follow‐up. Participation in the follow‐up phase was voluntary and uncompensated, leading to a smaller, potentially biased subsample. As a result, the longitudinal analyses were underpowered and less generalisable. Long‐term assessments are crucial for understanding the persistence of intervention effects and for informing practical recommendations, such as retraining intervals—in cases where more long‐term/persistent ST myth reduction is needed such as within professions with victim‐facing roles. Interestingly, the available data indicated that some positive effects became more pronounced over time, suggesting that delayed or cumulative impacts may warrant exploration in future research. Replicating this study with a larger and more heterogeneous sample across a longer timeframe would be an important next step in clarifying whether the lack of significant short‐term effects was due to an ineffective intervention or to ceiling effects in the present sample.

Although participants' guilt verdicts were measured as an outcome variable, the ecological validity of the experimental paradigm was limited in relation to real‐world juror decision‐making. The study's design differed substantially from an actual jury setting due to several factors, including the absence of juror eligibility screening, the lack of group deliberation, and the omission of comprehensive judicial instructions. These deviations constrain the extent to which the findings can be generalised to authentic courtroom contexts. Therefore, further research examining the impact of educational interventions on juror decision‐making, using a more ecological robust mock‐juror paradigm is warranted.

The present study only used one presenter for the intervention videos. Although manipulation checks indicated that most participants perceived the presenter to be credible and knowledgeable on the topic it, the authors cannot rule out whether characteristics specific to the presenter—such as age, occupation, gender or dialect—could have had a confounding effect on the impact of the intervention for some participants. Another fruitful direction for subsequent studies is to examine whether presenter characteristics can moderate the efficacy of such educational interventions (i.e., using different presenters in a between‐groups design).

Finally, much of the existing knowledge on ST myths derives from qualitative research (e.g., Rajaram and Tidball 2018). While such approaches have been instrumental in identifying key misconceptions, researchers have yet to translate this knowledge into a robust quantitative measurement tool. The Sex Trafficking Attitudes Scale (Houston‐Kolnik et al. 2016) captures certain ST myths within its broader attitudinal framework but remains limited in scope. Although Q. C. Miller et al. (2023) developed a more comprehensive measure, it has not yet been validated through confirmatory factor analysis or similar procedures. The present findings therefore reinforce the urgent need to develop and validate a psychometrically sound instrument capable of reliably capturing the full range of prevalent ST myths.

## 6 Conclusion

ST for the purpose of commercial sexual exploitation remains a pervasive global issue. Effective responses to combat trafficking and support victims depend on improving public understanding and dispelling harmful misconceptions. The current study did not find significant reductions in ST myth acceptance following exposure to an educational intervention video. It remains unclear whether this outcome reflects limitations in the intervention design or the highly pro‐victim stance of the sample, which may have constrained measurable improvement. Nonetheless, a modest protective effect was observed when comparing intervention and control participants, suggesting that even brief educational efforts may help preserve empathetic and informed attitudes over time. Further research is required to determine how educational interventions can most effectively reduce ST myths and foster attitudinal change. What is clear from the wider body of evidence is that reducing the acceptance of sexual violence myths—whether relating to rape or trafficking—is essential to improving victim support, protection, and justice outcomes.

## Author Contributions

Project conceptualization: Dara Mojtahedi. Literature review: Dara Mojtahedi, Gemma Hewitt, Sophie Fitton. Methodology: Dara Mojtahedi. Data collection: Gemma Hewitt, Sophie Fitton. Data analysis: Dara Mojtahedi. Interpretation of findings/discussion: Dara Mojtahedi. Manuscript draft writeup: Dara Mojtahedi, Gemma Hewitt, Sophie Fitton. Manuscript editing: Dara Mojtahedi.

## Funding

The authors have nothing to report.

## Ethics Statement

The authors received ethical approval from their institutional research ethics committee.

## Conflicts of Interest

The authors declare no conflicts of interest.

## Supporting information


Supporting Information S1

## Data Availability

The data that support the findings of this study are available on request from the corresponding author. The data are not publicly available due to privacy or ethical restrictions.
