# Supplementary material for: Sex Trafficking Myth Reduction: Evaluating an Educational Approach to Reducing Victim Blaming and Increasing Victim Empathy
Source: Behav Sci Law. 2025 Dec 20;44(2):181–92. doi: 10.1002/bsl.70034 (PMC13053908; doi:10.1002/bsl.70034)
Supplement: Supplementary file 1 — Supporting Information S1 [file BSL-44-181-s001.docx]

**Supplementary material (Experiment Vignette)**

***The following extract describes an incident where a female claims to have been coerced into prostitution***

Stephanie (21 years of age) was arrested for engaging in sexual activity in a public place and possession of a controlled substance (cocaine). The arrest occurred after a member of public made a complaint to staff, who then entered the bar toilets and interrupted Stephanie, and an unidentified male engaged in sexual intercourse. The staff informed them the police had been called.

During her initial interview at the station Stephanie disclosed that her partner, Ryan (38) was sexually exploiting her for financial gain, indicating it was Ryan who had made the arrangement between Stephanie and the unidentified male. Stephanie first encountered Ryan when she was 15 years old at a local bar: Stephanie would occasionally run away from home and would spend her evenings in various bars that didn’t ID her. She met Ryan at one of these bars and would occasionally talk to him. The two did not interact again until four years later when they ran into each other at a different bar. On this occasion, the two ended up going back to Ryan’s house and engaging in sexual activity. They continued to see each other and were in a relationship shortly after. Stephanie moved in with Ryan and his two children from a previous relationship. Stephanie helped around the house and looked after the children whilst Ryan worked various jobs. According to Stephanie, the exploitation started 4 months into their relationship. Below is an extract from Stephanie that details how the alleged abuse started.

**Stephanie's testimony:**
“ We would go to a few bars and clubs on Saturday nights, it was our ‘date night’. One night we were at this bar that was more of a snooker hall. After a few drinks, Ryan came up to me and told me that he wanted me to have sex with his friend in the toilets. I was angry and said ‘absolutely not!’ but he kept on persisting, he kept asking me to do him this favour just once. He continued to hassle me for at least thirty minutes, it made me very uncomfortable. He didn’t become aggressive or threaten me but it was clear that he wasn’t going to let this go so I eventually agreed and had sex with his friend. I was really disappointed the next week, he said that he was sorry but said that we needed the money to pay to survive and it was something I would have to occasionally do to help out. He did same thing two weeks later. Again, I said no but he kept pressuring me and I knew how this was going to end so I reluctantly agreed and went back to the stranger’s house for sex.

The next day he gave me some money and told me that this was the only way we could afford to survive but that I would not have to do it for long. Before I knew it, our Saturdays had changed. We were going to bars and getting drunk, then I would be told to have sex with a random person. I told him several times that I didn’t want to do this anymore and he would scream in my face, he threatened to kick me out of the house and he would call me horrible things.

Before all of this happened, I would occasionally have cocaine on a night out for a bit of fun. When he began making me have sex with random men, I found myself taking cocaine on most nights, just to get me through it. He gives me the cocaine because he knows it will make me do what he wants. I am not addicted or anything, but I need it to put up with the shit he has me doing. I feel disgusting and that he is ruining my life, but I have no money or anywhere to go

 **Ryan's Testimony:**

Ryan was interviewed following Stephanie’s allegations that he has been sexually exploiting her for financial gain. Ryan denies these allegations, claiming that their relationship involves a consensual agreement that they both benefit from, which includes Stephanie meeting with people Ryan selects. Below is an extract from Ryan’s account of their relationship and the events in question:

“Stephanie and I have been together for a while, and we have a bit of an arrangement that works for us. Sometimes, she’ll meet with people I set up, and there’s money involved. It’s  something we’ve done to keep things exciting. It’s always done in private which isn’t against the law. It’s not like I’m making her do it, she’s always keen to do it for the buzz, and we both understand that it helps us out financially. Stephanie’s had a rough time with her family, so she doesn’t really have anyone else to rely on. I know our relationship can get heated but I have never laid a hand on her. I’ve been the one looking after her and giving her a place to live, making sure she’s safe. As for the coke, yeah, we’ve used it here and there, mostly on nights out. It’s not something I’m proud of, just something we’ve done occasionally. If anything she takes it a bit more than me but she’s her own person.”
